# Beyond Inflammation - A Multidisciplinary Approach to Managing Obesity and Cardiometabolic Risk in Inflammatory Bowel Disease

**DOI:** 10.1007/s11892-026-01627-4

**Published:** 2026-06-18

**Authors:** Samantha L. Plush, Peter Litwin, Sangwoo Han, Robert V. Bryant, Saravana Kumar, Patricia Kaazan, Alice S. Day

**Affiliations:** 1 Department of Gastroenterology and Hepatology, The Queen Elizabeth Hospital, 28 Woodville Road, Woodville south, Woodville South, SA 5011 Australia; 2 School of Medicine, Adelaide University, 4 North Terrace, Adelaide, SA 5000 Australia; 3 Basil Hetzel Institute, 37 Woodville Road, Woodville South, SA 5011 Australia; 4 School of Allied Health and Human Performance, Adelaide University, Corner of North Terrace and Frome Road, Adelaide, SA 5001 Australia; 5 Department of Gastroenterology, Flinders Medical Centre, Flinders Dr, Bedford Park, 5042 SA Australia

**Keywords:** Inflammatory bowel disease, Obesity, Multidisciplinary team, Metabolic dysfunction-associated steatotic liver disease, Cardiovascular disease, Dietary management

## Abstract

**Purpose of Review:**

Adults with inflammatory bowel disease (IBD) demonstrate a high prevalence of obesity, metabolic dysfunction-associated steatotic liver disease (MASLD) and cardiometabolic comorbidities. Cardiovascular disease (CVD) is a leading cause of mortality in IBD. This review examines cardiometabolic and obesity screening, and management within IBD models of care to optimise chronic disease management.

**Recent findings:**

Obesity prevalence in IBD has increased to 40%. Chronic systemic inflammation driven by visceral adiposity, proinflammatory cytokine production, insulin resistance and vascular endothelial dysfunction represent a unifying mechanism linking IBD, MASLD and CVD. Proactive management of cardiometabolic risk is not prioritised within traditional IBD service models. Therapeutic approaches to manage obesity and lower CVD risk in this cohort with concurrent optimisation of IBD control include dietary and lifestyle intervention through to anti-obesity pharmacotherapy and bariatric procedures. Integrated multidisciplinary models of care that leverage existing infrastructure and shared-care partnerships with primary care, proactive risk identification and management strategies should be adopted within specialist IBD services.

**Summary:**

As obesity, MASLD and CVD emerge as major contributors of morbidity and mortality in IBD, failure to systematically address cardiometabolic risk represents a critical gap in contemporary IBD care. Embedding proactive screening and multidisciplinary management within existing IBD infrastructure offers immediate actionable opportunity to improve long-term outcomes beyond inflammatory control.

**Supplementary Information:**

The online version contains supplementary material available at 10.1007/s11892-026-01627-4.

## Introduction

Inflammatory bowel disease (IBD) encompassing Crohn’s disease (CD) and ulcerative colitis (UC) are immune mediated diseases with chronic relapsing and remitting inflammation mainly of the gastrointestinal tract [1]. Although undernutrition was previously the leading metabolic and nutritional disease-related complication of IBD (affecting 13.7–16.8% of non-admitted ambulatory patients), contemporary IBD cohorts show a high prevalence of obesity (15–40%) and metabolic syndrome (19%) [2–5]. Cardiometabolic risk is elevated in IBD with increased relative risk (RR) of ischemic heart disease (RR, 1.35; 95% CI: 1.19–1.52), cerebrovascular accident (RR, 1.25; 95% CI: 1.08–1.44) and coronary artery disease (RR, 1.17, 95% CI: 1.07–1.27) [6, 7]. The presence of metabolic dysfunction associated steatotic liver disease (MASLD) increases mortality risk in IBD, compared with IBD alone (HR, 1.58, 95%CI: 1.07–2.32) [8].

Obesity, cardiometabolic health and MASLD have not traditionally been prioritised within IBD care models, often addressed reactively rather than proactively as modifiable determinants of long-term IBD outcomes [9]. Management of these lifestyle-related diseases in IBD is complex but of prime importance as cardiovascular disease (CVD) has emerged as a leading cause of mortality in this cohort [10–12]. Early identification of metabolic risk facilitates timely intervention of modifiable parameters to reduce the progression of CVD and liver disease [13]. However, weight and cardiometabolic risk management in IBD is constrained by inadequate tertiary service infrastructure alongside disease-related challenges often experienced by people living with IBD including relapsing inflammation, corticosteroid use, surgery, impaired mental health and dietary complexities [14, 15].

A critical gap exists between the increasing burden of obesity and cardiometabolic disease in IBD and multidisciplinary frameworks to address these risks within routine IBD care. As such, this review examines cardiometabolic risk and obesity screening and management within IBD models of care to optimise chronic disease management in this cohort.

## Obesity and Poorer IBD Outcomes

Obesity is defined as a systemic relapsing chronic disease attributed to the presence of excess adiposity, resulting in altered function of tissues, organs or the entire individual [16]. Beyond energy storage and endocrine functionality, adipose tissue has immunological, metabolic and regulatory functions [17]. Visceral adipose tissue is considered a metabolically active organ housing multiple immune cells including proinflammatory macrophages and leading to upregulated production of adipocytokines including Interleukin-6 (IL-6) and Tumour Necrosis Factor Alpha (TNF-α). These cytokines activate inflammatory signalling pathways which are key therapeutic targets in IBD [18]. This dysregulation is proposed as a mechanism by which excess adiposity interferes with response to IBD therapy, and is associated with increased risk of surgery (adjusted risk (AR) 2.02, 95%CI: 1.09–3.76) and penetrating CD (AR 1.95, 95%CI: 1.04–3.67) in a cohort with visceral adiposity but where 50% classified as obese (BMI>30 kg/m^2^) [4, 17, 19]. Additionally, elevated visceral adipose tissue index (VATI), a quantified measure of visceral adipose tissue (> 18.42 cm²/m²) and mesenteric fat index (MFI) (> 0.87 cm²/m²) have been associated with intravenous corticosteroid treatment failure, a rescue therapy in CD [19, 20]. Obesity in patients with IBD represents a growing public health concern as the global burden of IBD is projected to exceed 1% of the population within the next decade, with morbidity compounded by a 2.5 fold higher prevalence of obesity in this cohort [21–23].

## Inflammation Driving IBD, Obesity, MASLD and CVD Risk

**Fig. 1:**
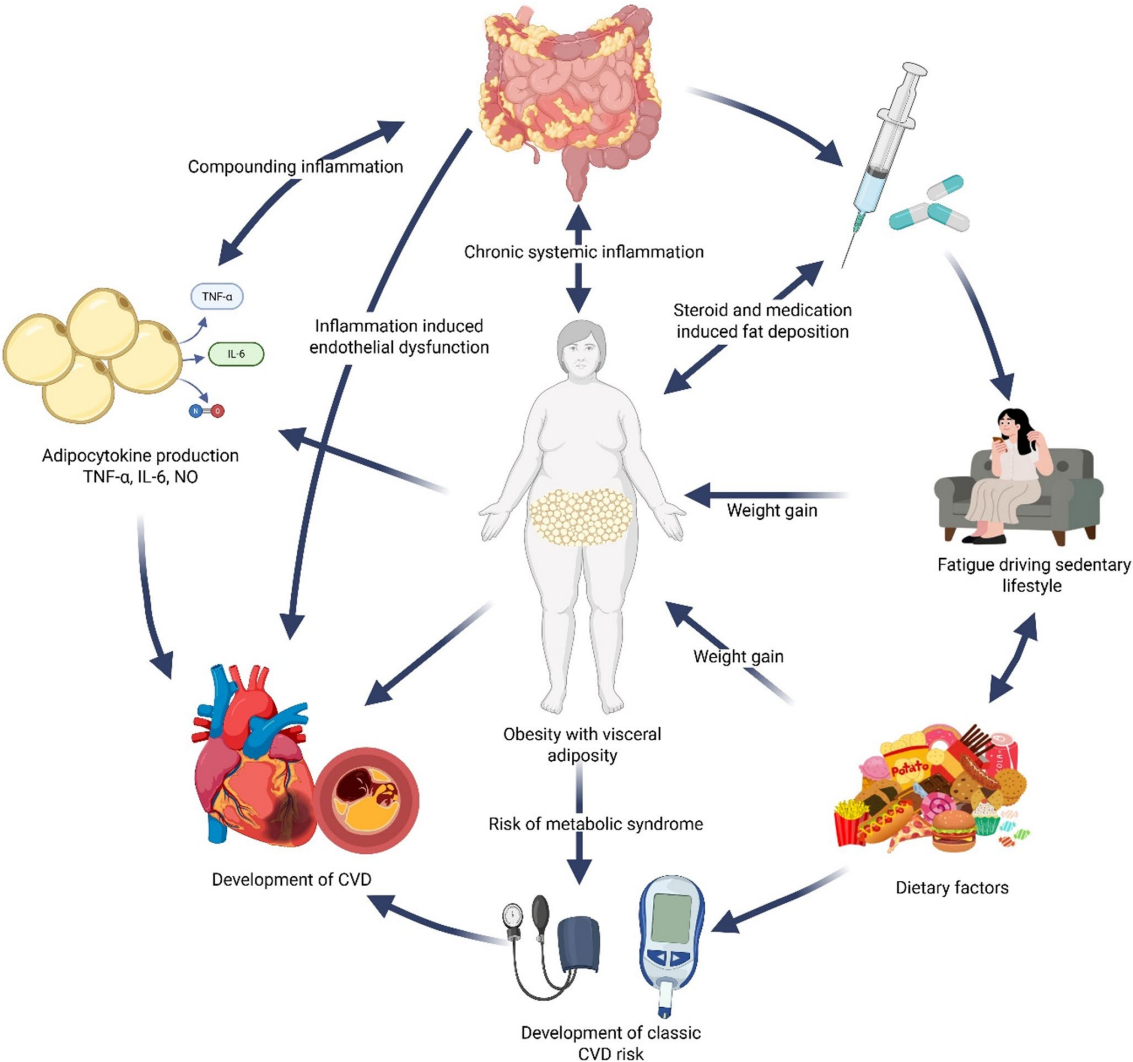
The complex interplay of risk factors driving visceral adiposity and cardiometabolic risk for patient with IBD

The relationship between IBD, MASLD, obesity and CVD is complex, bidirectional and multifactorial with chronic systemic inflammation representing a common pathway (Fig. 1) [24]. IBD is associated with increased rates of CVD (HR 1.98 (95%CI: 1.74–2.25)) compared to case-matched controls [25]. The elevated risk of CVD in the absence of classic cardiovascular risk factors such as hypertension, dyslipidaemia and Type 2 Diabetes Mellitus (T2DM) is thought to be driven by increased systemic inflammation [25, 26]. Perturbations in gastrointestinal microbiota, including a loss of diversity, are a shared feature in both IBD and obesity [27]. Alterations to bacterial community structure and function are associated with reduced metabolism of short-chain fatty acids (SCFA), impaired function of gastrointestinal endothelial barrier, increased permeability and translocation of commensal bacteria [27]. The ensuing low-grade inflammation is characteristic of obesity and other metabolic syndrome phenotypes driving CVD [24, 26, 27].

Immune dysregulation in IBD leads to upregulation of the aforementioned proinflammatory cytokines, TNF-α and IL-6, with increased nitric oxide (NO) and C reactive protein (CRP) production [24]. Elevated levels of cytokines observed in IBD are implicated in development of vascular endothelial dysfunction and resulting in atherosclerosis [24]. Additionally, both endothelial dependent and independent vasodilation is impaired in active IBD with high-sensitivity CRP significantly predicting the degree of endothelial dysfunction [28]. Conversely, effective treatment of active IBD reduces systemic inflammatory burden and is associated with reduced rates of CVD, forming a biological rationale for treat to target strategies in IBD management [24].

Chronic systemic inflammation is also implicated in the pathogenesis of MASLD and amplification of cardiometabolic risk in IBD [19, 29, 30]. Excess visceral adipose tissue disrupts hormonal and metabolic homeostasis, contributing to resistance of insulin sensitive cells and dysregulated lipid metabolism driven by infiltration of inflammatory cells in adipose tissues [19, 31]. Skeletal muscle insulin resistance leads to further decreased muscular glucose uptake, hyperinsulinemia, and greater free fatty acid circulation accompanied by increased reactive oxygen species and greater proinflammatory adipocytokine production [32]. Hepatic insulin resistance leads to hepatocytes shunting excess glucose into lipogenic pathways, thus contributing to hepatic steatosis and MASLD development [32]. A diagnosis of IBD is an independent risk factor for advanced liver fibrosis associated with MASLD (aOR 1.99 *p* < 0.001) which translates to a greater risk of mortality (HR 1.58 95% CI: 1.07–2.32) compared with patients with IBD without MASLD [8].

## Iatrogenic Cardiometabolic Risk Across IBD Therapy

Corticosteroid exposure represents an iatrogenic contributor to cardiometabolic risk in IBD [33]. Prolonged steroid use (> 3 months) is associated with body weight increases of between 5 and 13%, compounded by repeated steroid courses [34]. Systemically, corticosteroids impair hepatic insulin sensitivity while reducing glucose uptake in skeletal muscle and adipocytes, promoting hyperglycaemia and insulin resistance [34, 35]. Compounding metabolic risk, insulin resistance and resultant hyperglycaemia stimulate a defensive inflammatory cascade leading to increased cytokine levels, particularly in the liver, which drive further fatty acid mobilisation forming a lipotoxic environment and contributing to both increased CVD and MASLD risk [36]. In a large IBD cohort, each 5 mg increase in daily corticosteroid was associated with a 7% increase of all-cause CVD (HR of 1.07 95%CI: 1.06–1.09) [37]. Systemic corticosteroids remain widely used as an effective induction therapy despite the potential long-term metabolic complications [33].

Immunomodulator therapy has also been associated with body composition changes over 24-months, with increased fat mass index 0.33 kg/m^2^ (95% CI: 0.14–0.53) and subsequent reduction in lean muscle mass (appendicular skeletal muscle index − 0.07 kg/m^2^, (95% CI: −0.12- −0.01) observed in 110 adults with IBD (72% CD) [30]. Following IBD induction therapy such as anti-TNF-α monoclonal antibodies, many adults gain weight [38]. After 48 months of infliximab therapy, adults (*n* = 165) gained a mean of 2.4 kg (95% CI: 1.4, 3.4) and an associated body mass index (BMI) gain of 0.77 kg/m^2^ (95% CI: 0.43, 1.1) [38]. Lower baseline BMI, male sex, higher CRP and lower album levels at baseline were associated with increased rates of weight gain [38]. Unfavourable shift in body composition is likely a result of improved inflammation, nutrient absorption and reduced gastrointestinal symptoms that impair appetite during a flare rather than driven by the medical therapy, yet equally contributes to metabolic risk [19, 30, 39].

The introduction of Janus Kinase (JAK) inhibitors and Sphingosine 1-phosphate (S1P) receptor modulators as advanced therapies in IBD has further complicated CVD risk profiling [40, 41]. IBD expert guidance recommends screening of CVD risk factors and risk stratification prior to initiation of JAK inhibitors or S1Ps [40]. S1Ps have been associated with new onset hypertension [40]. While JAK inhibitors are associated with modest increases in total cholesterol, high-density lipoprotein cholesterol (HDL-C), and low-density lipoprotein cholesterol (LDL-C), the LDL-C to HDL-C ratio is typically stable [40]. Reassuringly these lipid changes appear reversible with statin treatment [40]. In the ORAL surveillance study, a higher risk of major adverse cardiovascular events (MACE) was observed in older adults with rheumatoid arthritis receiving tofacitinib compared with TNF-α inhibitors [41]. In contrast, the incidence of MACE in IBD populations treated with tofacitinib was low (Incidence rate (IR) 0.29 (95%CI: 0.13–0.55), and comparable rates observed with anti-TNF agents in patients with UC (IR 0.51, 95%CI: 0.31–0.79) [40]. Pre-existing atherosclerotic CVD consistently characterised patients who developed MACE on JAK inhibitors, further underscoring the importance of baseline cardiovascular risk assessment prior to advanced therapy commencement [40].

## Screening for Obesity and Cardiometabolic Risk in IBD Clinic

Proactive early identification of visceral adiposity and CVD risk within integrated care is an obvious strategy to prevent progression to CVD and cirrhosis and reduce healthcare burden [17, 20]. However, there is a paucity of clinical guidance to inform the optimal risk screening commencement and frequency for adults with IBD. In Table 1, key components of interdisciplinary cardiometabolic and obesity related risk screening are outlined. Routine screening to detect and monitor CVD risk in adults with IBD appears prudent for most assessment domains presented in Table 1 [4, 42, 43]. Increased frequency of screening is recommended for higher risk populations within the IBD cohort including indigenous populations and those with a strong first-degree family history [44, 45].

Assessing cardiometabolic risk requires attention to more than weight-based indices alone, as metabolic syndrome exists in adults with a healthy BMI, especially for those with IBD [24, 46]. BMI requires clinical interpretation as it describes body mass quantity rather than quality [47]. Waist to height ratio is a more accurate predictor of body composition to routinely record alongside BMI in adults with IBD as it strongly correlates with visceral adipose tissue area measured on MRI or CT [16]. Routine biochemical and clinical evaluation of metabolic syndrome biomarkers, seen in Table 1, can be incorporated as part of standard IBD biochemical monitoring.

Outpatient appointments, biologic monitoring and flare follow-up provide pragmatic opportunities to embed CVD screening into IBD clinics. Clear role delineation and documentation between outpatient nursing staff, IBD nurse specialists, gastroenterologists and the primary health care team provides continuity of care. Embedding systematic, role-defined screening within existing IBD workflows offers a scalable approach to reducing long-term cardiometabolic risk in this population at high-risk of CVD.

**Table 1:** Summary of recommended cardiometabolic, nutritional, functional, and lifestyle screening domains relevant to adults with IBD to inform integrated cardiometabolic risk prevention strategies

Assessment Domain	Screening Tools	Screening Onset in IBD	Frequency	Relevance to IBD	Implementation Notes	Clinician to Complete Screening	Source of Recommendation
Cardiometabolic Panel	Blood pressure, Lipid profile, Fasting glucose	Age > 30 years without specific risk factors Prior to advanced therapy initiation [42, 43, 48]	Yearly, informed by risk factors and prior results [42, 43, 48]	S1P receptor modulators elevate blood pressure JAK inhibitors increase HDL and LDL cholesterol Fasting glucose levels elevated with corticosteroids [49, 50]	Blood pressure monitoring during biologics screening. Lipids and fasting glucose can be included in routine IBD blood monitoring	PCP, IBD nurse specialist	RACGP guidelines for preventative activities in General Practice [43]
**Body composition and Functional Assessment**							
Body Composition Metrics	BMI, Waist circumference, Waist-to-height ratio	At service introduction	6-monthly to yearly or when composition metrics shift	Elevated BMI may impact disease course and medication efficacy. Visceral adiposity associated with complex Crohn’s phenotypes. Waist-to-height ratio predicts cardiometabolic risk. [19, 23, 51, 52]	Ethnic-specific normative values available BMI lacks granularity in assessing muscle versus fat mass [52]	PCP, Nurse, Dietitian	RACGP guidelines for preventative activities in General Practice [43]
Functional Assessment	Hand grip strength, Sit-to-stand test	At service introduction	Yearly or 6-monthly if risk detected on previous screening	50% of adults with IBD age > 35 years have reduced physical performance Age and gender-specific cutoffs available. Validated in IBD [14, 53]	Handgrip dynamometry is inexpensive and accessible. Sit-to-stand requires no specialist equipment [52]	Dietitian, IBD nurse specialist, Exercise physiologist	BDA consensus guidelines (nutritional assessment and dietary management of patients with IBD) [53] ECCO consensus on dietary management of IBD [54]
**Nutrition screening**							
Disordered Eating Assessment	SCOFF[55]	Based on clinical history and judgement		Disordered eating is prevalent in adults with IBD and or obesity [56, 57]	No validated tools for IBD. May overrepresent risk, increasing service burden. [55, 58]	Self-administered	Cohort study [58]
Malnutrition Risk	IBD IBD-NST [59] or MUST [60]	At service introduction	Annually or following major clinical events (flare/surgery)	Metabolic comorbidities and malnutrition risk coexist in IBD [59, 60]	Screens unintentional weight loss despite BMI and patient-reported nutrition concerns. Validated digital or self-administration [59]	Outpatient nurse, or self-administration	BDA consensus guidelines (nutritional assessment and dietary management of patients with IBD) [53] ECCO consensus dietary management of IBD [54]
**Lifestyle factors**							
Alcohol Intake	AUDIT [61]	At service introduction Prior to medication change with alcohol interaction	Yearly	Associated with increased GI symptoms in some. Potential pathological role in IBD [62]	Can use screening tools or incorporate into clinical assessment	All clinicians involved in patient care	WHO STEPs Manual [63, 64]
Physical Activity	IPAQ or GPAQ [65, 66]	At service introduction	Yearly	60% of adults with IBD are sedentary [67]	Screening questionnaires can be used prior to clinic attendance [68]	All clinicians involved in patient care	WHO STEPs Manual [63, 64]
Smoking Status	Clinical assessment	At service introduction Prior to advanced therapy initiation [49]	Yearly if non-smoker or at each encounter if smoker [49]	Associated with poorer IBD disease course and other risks [69]	Evidence that adults with IBD should be warned of continued smoking risks [49, 62]	All clinicians involved in patient care	WHO STEPs Manual [63, 64]
**Mental health**							
Mental Health	PHQ-9 [70]	At service introduction	Yearly or following major clinical events (flare/surgery) [71]		Tools inform risk presence indicating progression to formal diagnosis. Access to psychologists can be a barrier in IBD centres [15]	All clinicians involved in patient care	Cohort and validity studies [71]

Table 1 footnotes: The SCOFF questionnaire is a 5 question screening tool for disordered eating [55]. The Inflammatory Bowel Disease-Nutrition Screening Tool (IBD-NST) is a 4 question screening tool to assess nutrition risk in IBD [59]. The Malnutrition Universal Screening Tool (MUST) is a 4 step screening tool used in diverse patient populations, including IBD [60]. Alcohol Use Disorders Identification Test (AUDIT) is a validated 10 question screening tool to identify alcohol use disorders [61]. The Exercise International Physical Activity Questionnaire (IPAQ) is a set of 4 questionnaires on health-related physical activity [66]. The Global Physical Activity Questionnaire is a set of 16 questions covering several components of physical activity including duration, intensity and frequency (GPAQ) [65]. The Patient Health Questionnaire (PHQ-9) is a 9 item questionnaire assessing the presence of and severity of depressive symptoms [70]. *BMI *body mass index, *BDA* British dietetics association, *CT *computer tomography, *ECCO *European crohn’s and colitis organisation, *GI *gastrointestinal, *HDL *high density lipoprotein, *IBD *inflammatory bowel disease, *JAK* janus kinase inhibitor, *LDL *low density lipoprotein, *MRI *magnetic resonance imaging, *OPD* outpatient department, *PCP *primary care physician, *RACGP* Royal Australian college of general practitioners, *S1P *sphingosine-1-phosphate, *WHO* World Health Organisation

## Therapeutic Strategies for Obesity and Cardiovascular Risk Reduction in IBD

Obesity and visceral adiposity are modifiable risk factors for CVD, MASLD and IBD and can be targeted through diet and lifestyle modification, pharmacotherapy and surgical interventions (Supplementary Fig. 1) [16, 72]. Treatment is informed by clinical assessment, risk/benefit analysis and shared clinical decision making (Supplementary Fig. 1) [16]. International guidelines for management of obesity recommend a bottom-up approach with diet and lifestyle intervention at lower BMI thresholds and escalation to anti-obesity medications, and bariatric surgery or endoscopic bariatric therapies considered at higher BMI or in the presence of metabolic complications [73–75]. A summary of management strategies to support weight reduction in overweight or obese adults with IBD are found in Table 2 & Supplementary Fig. 1.

**Table 2:** Comparisons of weight management interventions with evidence to reduce weight and/or cardiometabolic risk in adults with inflammatory bowel disease

Intervention	Target population	Mechanism	Implementation in practice	IBD specific considerations	Results	Enablers in IBD	Barriers in IBD
**Dietary Interventions**							
Crohn’s disease exclusion diet (CDED) [80]	Mild - moderate CD CDED indicated as IBD therapy	50% PEN with 5 mandatory foods and a further 14 allowed foods to reduce to exposure proposed proinflammatory dietary components [106, 107]	12 weeks of CDED to provide between 30–35 kcal/kg/day	Stronger evidence for paediatric CD with small trials in adults [80, 107]	BMI decreased from 25.8 kg/m² (IQR 20.8–28.1) to 24.4 kg/m² (IQR 20.4–27.6; *p* = 0.007), versus no change with MedDiet 23.0 kg/m² (IQR 21.7–26.3) to 23.0 kg/m² (IQR 20.7–27.1; *p* = 0.97) Fat mass decreased (18.2% vs. 15.5%; *p* < 0.0001), lipid profile unchanged. [80]	Allowance of food compared with EEN proposed to increase adherence [106, 107]	Requires multidisciplinary team and IBD dietitian to minimise any unnecessary dietary restriction [108]
Exclusive enteral nutrition [79]	Active CD EEN indicated as IBD therapy	Yet to be elucidated, Calorie deficit can be achieved while meeting other micronutrients and protein requirement [109]	8 weeks of EEN personalised prescription in non-obese adults [79] Optimal care pathways for implementation and monitoring [110]	Monitoring of adherence should occur with an IBD dietitian [52]	VFA was decreased after EEN compared with baseline median 83.2 (IQR 44.36, 112.34) versus median 93.88 (IQR 45.63, 124.23 *p* > 0.03) [79]	Cost is comparable to food costs. Monitoring of adherence with an IBD dietitian [52]	Limited access to indirect calorimetry to personalise EEN prescription.
Mediterranean diet [111]	Any IBD type with a BMI < 30 kg/m^2^ [112]	Increased consumption of phytonutrients, polyphenols and vitamins [113]. High quality diet replaces low micronutrient density, high caloric intake. [81]	Inclusion of a wide variety of foods, such as extra-virgin olive oil, legumes, cereals, nuts, fruits, vegetables, dairy products, fish, coupled with reduction in red meats and discretionary foods [113].	Increased bulking fibre, care required in patients with stricturing disease [114]	6 months of adherence led to improved BMI (UC − 0.4, *p* = 0.002; CD − 0.5, *p* = 0.03) and waist circumference (UC − 1.3 cm, *p* = 0.04; CD − 1.4 cm, *p* = 0.041) [111]	Adherence associated with female sex, older age (> 62 years), moderate alcohol consumption, and implementation by dietitian [52, 115] Cost-effective compared to typical Western diets [112]	Lower adherence associated with lower education levels, higher waist-to-height ratio, diabetes, less physically active, single or separated relationship status, and smokers [115]
**Physical activity**							
Moderate intensity aerobic and resistance exercise [88]	Beneficial for all	Improved lean muscle increases metabolic requirements Associated with anti-inflammatory and multi-organ metabolic effects [116, 117]	8-week intervention based on couch to 5 km training program 3x weekly training sessions within gym with supervision from personal trainer	Fatigue impacts exercise adherence, but exercise is demonstrated to improve fatigue scores in IBD [67]	Decreased total body fat percentage of −2.1% (−2.1, −0.5) versus a gain of + 0.1% (−0.04,1) total body fat in the control group (*p* = 0.022)	App based programs increases access [118]	Cost of supervised exercise programs for IBD Fatigue [67]
**Anti-obesity medications**							
Semaglutide	BMI of ≥ 30 kg/m^2^ or ≥ 27 kg/m^2^ with metabolic comorbidities [119]	Enhancing glucose dependent insulin secretion, supressing glucagon, slowing gastric emptying and increasing central satiety [120]	Gradual increase in doses to reduce gastrointestinal side effects [120] Counselling on potential transient side effects to increase adherence [121]	Good safety profile, no increased risk of steroids use and lower risk of all cause hospitalisation (OR 0.4 95%CI: 0.2–0.7 *p* = 0.001) [93]	Weight loss (*n* = 442) of − 9.1 kg (95% CI: −11.8; −6.4, I2 = 12%, *p* < 0.0001) [122]	Multidisciplinary approach including dietary and lifestyle advice [123]	Cost may be prohibitive Discussion surrounding lifelong use of anti-obesity medications for most [123]
Tirzepatide	BMI of ≥ 30 kg/m^2^ or ≥ 27 kg/m^2^ with metabolic comorbidities [119]	GIP further enhances glucose dependent insulin secretion, modulating adipocyte metabolism and central appetite pathways [92]	Doses of > 15 mg tirzepatide induced more weight loss than doses 5–10 mg in IBD [119] Counselling on potential transient side effects to increase adherence [121]	Similar risk of steroid use, advanced therapy initiation, raised faecal calprotectin or CRP and ED visit compared with matched controls [92]	Weight loss of − 11.6 kg, 95% CI: −18.3; −4.8, I2 = 0%, *p* = 0.0008, *n* = 113) [122] Weight loss trajectory independent of IBD medical therapy [92]	Multidisciplinary approach including dietary and lifestyle advice [123]	Cost may be prohibitive Discussion surrounding lifelong use of anti-obesity medications for most [123]
**Surgical management**							
Bariatric surgery	BMI ≥ 40 kg/m^2^ or a BMI ≥ 35–40 kg/m^2^ with metabolic comorbidities [73]	Varied surgical techniques designed to reduce gastric volume or bypass key nutrient absorption sites [73]	SG or RYGB have a pooled reduction in BMI of 13.7 kg/m^2^ (95%CI: 12.5–14.9) [103]. However, SG showed trends toward less adverse events being considered intestinal sparing [103].	Similar risk of intravenous and oral steroid use, advanced therapy initiation, raised faecal calprotectin or CRP and ED admissions compared with propensity matched IBD controls [92]	Loss of 29.3 ± 16 kg or 24.5 ± 12.7% TBW [124]. BMI reduction from 42.1 ± 7.07 kg/m^2^ to 34.4 ± 6.9 kg/m^2^ after 1 year [125] Decrease in IBD medications in 45.6% post-surgery (95%CI: 23.8–69.2) [103]	Outperforms oral obesity medications including Tirzepatide [92] Bariatric surgical predictive tools are available to support risk/benefit decision making [126].	Specialist bariatric IBD centres required [73]. Limited access to multidisciplinary dietary and lifestyle intervention.
Endoscopic Bariatric surgery	BMI ≥ 30–40 kg/m^2^ [127]	Multiple endoscopically delivered techniques to restrict gastric lumen volume, or reduce contact of nutrients with the small intestinal mucosa impairing absorption [127]	Intragastric balloon and endoscopic SG assessed in adults with IBD [96]	Limited observational data [96, 101, 102].	Reduction of BMI from 46.8 kg/m^2^ to 35.6 kg/m^2^ over a 6 month period [96]	Multidisciplinary approach which including dietary and lifestyle advice [73, 127]	Specialist interventional endoscopist services required [96]

Footnotes: Elevated faecal calprotectin is defined as ≥ 250 µg/g and raised C reactive protein as ≥ 10 mg/l. *BMI *body mass index, *CD *crohn’s disease, *CDED *crohn’s disease exclusion diet, *DEXA *dual energy Xray absorptiometry, *ED* emergency department, *EEN *exclusive enteral nutrition, *GIP *glucose-dependent insulinotropic polypeptide, *GLP-1 *glucagon-like peptide-1, *MedDiet *mediterranean diet, *MFI* mesenteric fat index, *PEN* partial enteral nutrition, *RYGB *roux-en-Y gastric bypass, *SD *standard deviation, *SG *sleeve gastrectomy, *TBW *total body weight, *UC *ulcerative colitis, *VFA *visceral fat area

### Dietary Strategies

Dietary therapy can contribute to inflammation control, maintenance of remission and improving outcomes in IBD, while also recognised to have a central role in the management of MASLD and CVD [54, 76, 77]. However, evidence for dietary interventions which simultaneously address IBD activity, improve body composition and metabolic comorbidities remain limited (Table 2). Adherence to healthy eating patterns can confer cardiovascular protection; however, more prescriptive dietary therapies appear necessary to influence IBD driven inflammation [54, 77].

### Dietary Interventions in Crohn’s Disease

Exclusive enteral nutrition (EEN) is an established short-term induction therapy for CD [78]. In an observational cohort (*n* = 38), visceral and mesenteric fat indices were reduced following 8-weeks of EEN independent of weight stability, Table 2 [79]. Crohn’s disease exclusion diet (CDED) may be considered for induction therapy in CD. In an open-label randomised control trial (RCT) of 24 adults with CD, 12 weeks of CDED reduced BMI compared with the Mediterranean diet (MedDiet), while fat-free mass increased, Table 2 [80]. Unlike the MedDiet, CDED did not improve lipid parameters, limiting its cardiometabolic benefit beyond weight loss [76, 80]. Dietary composition in the MedDiet may drive favourable body composition changes beyond reduced caloric prescription alone, this evidence within the literature is methodologically limited, highlighting the need for further high-quality research [78, 80].

### Dietary Interventions in Ulcerative Colitis

Diet therapies are positioned to support maintenance of remission of UC, however current evidence is insufficient to recommend diet as a monotherapy for induction of UC remission [54]. MedDiet may be considered as an adjunct to medical therapy for UC maintenance with associated improvements in weight and BMI, Table 2 [54]. In non-IBD cohorts greater MedDiet adherence is associated with reduced CVD risk (RR 0.70 (95% CI: 0.62–0.80)) energy-restriction, and improved anthropometric outcomes [76, 81]. Comparative studies demonstrate culturally adapted healthy diet patterns improve body composition, yet only MedDiet provides cardiovascular protection [82]. Given strong evidence supporting MedDiet in improving cardiometabolic risk and MASLD, it may be considered for most adults with IBD and concurrent metabolic risk with implementation overseen by an IBD dietitian to individualise to IBD specific requirements, disease activity and phenotype [52, 83].

### Dietary Supplementation

Curcumin, a medicinal plant commonly formulated as a dietary supplement exhibits metabolic and anti-inflammatory effects [84]. In non-IBD cohort, nano-curcumin supplementation reduced CRP (WMD: −1.29 mg/L; 95% CI: −2.15 to − 0.44; *p* = 0.003), fasting glucose levels (WMD: −1.21 mg/dL; 95% CI: −1.43 to − 1.00; *p* < 0.001) and Homeostatic Model Assessment for Insulin Resistance (HOMA-IR) (WMD: −0.28 mg/dL; 95% CI: −0.33 to − 0.23; *p* < 0.001) while increasing HDL (WMD: 5.77 mg/dL; 95% CI: 2.90 to 8.64; *p* < 0.001) [84]. ECCO guidelines suggest curcumin may be used as an adjunct therapy to mesalamine for induction of remission of mild to moderate UC, while delivering potential concurrent benefits of improved cardiometabolic outcomes. Optimal dosage and formulation remain unclear and concerns regarding hepatotoxicity necessitate further research before routine clinical implementation [54].

### Physical Activity

Like non-IBD cohorts, 60% of adults with IBD do not meet recommend physical activity guidelines, with physical activity levels negatively correlated with fatigue (*r*=−0.224 *p* < 0.001) [67, 85, 86]. While evidence to guide physical activity to improve health outcomes in IBD is limited, physical activity remains a cornerstone to improving metabolic health [73, 87]. In adults with IBD, structured moderate intensity aerobic and resistance exercise improved body composition [88]. Similar benefits was observed in a MASLD cohort in addition to improving metabolic indices (5% reduction in intrahepatic triglyceride content, (− 1.64 (95% CI; −3.38 to 0.10)), reduced fasting insulin HOMA-IR (0.14 (95% CI: −1.48–1.76)) and blood pressure (− 5.48 mmHg (95% CI: −6.51, − 4.45; *p* < 0.001)) [89–91]. Combining resistance and aerobic exercise to the levels recommended in national guidelines has the potential to improve metabolic health [86, 87].

### Anti-Obesity Medications

Adjunct to diet and exercise, anti-obesity medications may be considered for promoting weight loss in patients with IBD [92, 93]. There are multiple ongoing RCTs investigating the role of Glucagon like peptide-1 receptor agonist (GLP-1 RA) for both weight management and an induction therapy in IBD (NCT06774079, NCT06937099, NCT06976853). International guidelines discourage prescription of older agents (Orlistat and Bupropion-Naltrexone) due to inferior efficacy and adverse effects, favouring GLP-1 RA [73]. Semaglutide and Tirzepatide have been reported to be associated with clinically meaningful weight loss, comparative to propensity matched non-IBD controls in retrospective cohort studies, Table 2 [92, 93]. A Danish registry based study found that GLP-1RA therapy was associated with improvements in IBD-related outcomes, including reduced steroid use and hospitalisation rates, in addition to weight loss, in patients with T2DM and IBD [94].

### Surgical or Endoscopic Bariatric Interventions

In IBD, bariatric surgery and endoscopic bariatric therapy (EBT) are safe modalities to reduce obesity and IBD related complications for those with BMI greater than 40 kg/m^2^ or BMI ≥ 35 kg/m^2^ with metabolic comorbidities [95–98]. Sleeve gastrectomy (SG), an intestine sparing procedure, is a common bariatric surgery in IBD demonstrating significant reductions in BMI, dosing of immunomodulators and reduction in steroid usage, Table 2 [97, 99]. EBT is an evolving field within interventional endoscopy with favourable risk benefit profile, but at the cost of efficacy [100]. Observational studies have demonstrated an adequate safety profile and efficacy of these procedures in adults with IBD [96, 101, 102].

### Monitoring Weight Loss in Adults with IBD

Greatest weight loss occurs within the first 12 months following bariatric surgery or initiation of pharmacotherapy, with a pooled BMI loss in IBD of 13.7 kg/m^2^ (95% CI: 12.5–14.9) following bariatric surgery and a mean BMI reduction of 2.48 kg/m^2^ (95% CI: 0.79–4.17) following commencement of GLP-1RAs [103, 104]. Structured nutritional counselling increases the likelihood of successful weight loss (OR 1.56, 95% CI: 1.02–2.38, *p* = 0.04) [105]. Multidisciplinary monitoring is essential to detect transition to unintentional weight loss, sarcopenia or treatment inefficacy [52, 54]. IBD specialist dietitians play a central role in monitoring and managing adverse effects and optimising long-term outcomes [52].

## Opportunities to Address Unmet Outient Needs

IBD multidisciplinary teams (MDTs) are ideally positioned to address obesity in people living with IBD, yet rarely integrate obesity and cardiometabolic management into routine IBD care [128]. Specialist IBD MDTs coordinate complex patient-centric bio-psycho-social longitudinal care and can contextualise obesity management within this demographic. Core MDT members include the gastroenterologist, colorectal surgeon, IBD nurse specialist, radiologist, dietitian, psychologist, pharmacist, and Primary care physicians (PCPs), with access as required to exercise physiologists, bariatric surgeons and obesity medicine specialists to enable management of cardiometabolic risk factors [129].

Integrating specialist IBD teams and primary care systems can position obesity and metabolic health as core rather than peripheral functions to offer evidence-based solutions [15]. The IBD nurse specialist anchors this model by coordinating risk profiling encompassing assessments tools discussed in Table 1, triggering appropriate MDT referral as indicated [130]. Adaption of “Intensive Medical Home” care models designed to address IBD activity provides an alternate nurse-led evidence-based approach to cardiometabolic risk screening. Medical Home models focus on MDT-based care, nurse coordination, tracking relevant IBD outcome metrics, and appropriate adoption of technology and after-hours support. This service model is primed to incorporate cardiometabolic risk assessment by leveraging existing biologic and safety monitoring infrastructure to achieve holistic care [131]. Medical Home establishment in a mixed IBD cohort has been associated with reduction in emergency department (ED) visits and hospitalisation (47.3% (*p* < 0.0001), 35.9% (*p* = 0.008) respectively [131]. Further, utilisation of technology results in efficient, effective care [132]. Web-based e-Health systems delivered a 5-fold reduction in hospitalisation in a cohort of 233 patients with UC (21 versus 107 acute visits *p* < 0.0001) [132]. Importantly in meta-analysis, e-health technologies are associated with improved quality of life metrics in adults with IBD (SMD 0.20 (95% CI: 0.05–0.35, *p* = 0.008) [133]. Both Medical Homes and e-health technology can be adapted to include chronic disease screening and digital management strategies for CVD [134, 135].

Nurse-led telemedicine has been shown to significantly decrease hospital admissions, IBD-related surgeries, associated comorbidities and reduce overall service healthcare costs [136]. However, persistent workforce shortages and inequitable access to allied health professionals constrain capacity to add routine obesity and cardiovascular risk management without service redesign [15]. Self-management resilience training has demonstrated reduced ED visits and hospitalisation (71% and 94% respectively) in the IBD cohort [137].

PCPs can provide comprehensive cardiometabolic risk assessment individualised preventative care to adults with IBD [138, 139]. Systematic data sharing between specialist IBD services and PCPs will facilitate integrated care through interoperable electronic records, embedded risk assessment tools and standardised referral templates to avoid duplication, support risk stratification, and enable gastroenterologists to consider obesity and cardiometabolic risk when making IBD treatment decisions [138]. Optimised digital platforms would strengthen collaborations with PCPs, community pharmacist and specialist IBD team, to significantly reduce clinical and economic burdens in the management of obesity, MASLD, IBD and CVD [138].

Implementation of an appropriate service model requires co-design with consumers, PCPs and specialist teams to clarify roles and ensure equitable access [139, 140]. Evidence from chronic disease management emphasises structured screening, self-management support, care coordination, and clear escalation pathways, elements that translate well to integration of obesity management in IBD [141, 142]. For example, in services unable to host full MDTs, formalised links with PCP-led primary health care encompassing community pharmacist, psychologists, exercise professionals, and obesity specialised clinics can fill expertise gaps, while preserving IBD teams as the coordinating hub for complex decision-making (Supplementary Fig. 2).

## Conclusions

The exponential increase of obesity, MASLD and CVD mortality in patients with IBD demands an urgent reorientation of IBD care models [10, 11]. Obesity and visceral adiposity affect up to 40% of adults with IBD, amplifying CVD risk through synergistic inflammatory pathway, while simultaneously compromising disease outcomes and therapeutic efficacy [19]. With CVD remaining a leading cause of mortality in IBD, the failure to systematically address obesity and cardiometabolic risk represents a critical gap in contemporary IBD care [10–12].

Across the therapeutic spectrum from MedDiet, GLP-1RAs, endoscopic interventions, and bariatric surgery, there are effective tools to modify obesity and cardiometabolic risk for patients with IBD [76, 92, 93, 125]. Early identification through systematic screening of BMI, waist circumference, and metabolic risk factors provides the foundation for early intervention [16, 48]. Reframing current technologies may enable opportunistic assessment and monitoring of cardiometabolic risk. Yet systematic evidence gaps persist, including optimal screening frequency in IBD and care pathways for managing obesity and cardiometabolic disease through the spectrum of lifestyle modification, pharmacotherapy to endoscopic therapies and conventional bariatric surgery for adults with IBD [28]. The integration of risk assessment into existing IBD infrastructure, leveraging biologic monitoring visits, nurse-led clinics and digital health platforms offers a pragmatic solution for immediate action [143–145].

Amid the global obesity pandemic, clinicians must urgently broaden their focus as patients with IBD aren’t exempt from the complications of obesity and cardiometabolic risk, which increasingly compounds disease complexity and worsens outcomes [17]. Reframing IBD care to focus on preventative strategies will safeguard metabolic health, reduce long-term cardiovascular risk and improve quality of life for millions of adults with IBD globally.

## Key References

- Pedersen SD, Manjoo P, Dash S, Jain A, Pearce N, Poddar M. Pharmacotherapy for obesity management in adults: 2025 clinical practice guideline update. Cmaj. 2025;197(27):E797-E809.
  - ○ This 2025 Canadian clinical practice guideline developed through systematic review and expert consensus, provides guidance on the use of obesity pharmacotherapy in a range of premorbid conditions including MASLD and CVD.
- Mahmoud M, Syn WK. Impact of Obesity and Metabolic Syndrome on IBD Outcomes. Dig Dis Sci. 2024;69(8):2741-53. doi: 10.1007/s10620-024-08504-8.
  - ○ This review explores the potential by which modification of obesity and associated metabolic comorbidities alter key IBD outcomes including treatment response, surgical rates, development of complications and health economics.
- Rubino F, Cummings DE, Eckel RH, Cohen RV, Wilding JP, Brown WA, et al. Definition and diagnostic criteria of clinical obesity. The Lancet Diabetes & Endocrinology. 2025;13(3):221 − 62.
  - ○ This paper reconceptualises obesity as a chronic disease rather than a peripheral risk factor providing a direct link between adiposity and organ dysfunction seen in MASLD and CVD. The commission provides clinically relevant evidence-based definitions for the diagnosis of clinical obesity.
- Svolos V, Gordon H, Lomer MC, Aloi M, Bancil A, Day AS, et al. European Crohn’s and Colitis Organisation consensus on dietary management of inflammatory bowel disease. Journal of Crohn’s and Colitis. 2025;19(9):jjaf122.
  - ○ This International consensus developed through systematic search strategy coupled with expert consensus, provides structured evidence-based dietary guidelines for IBD.

## Supplementary Information

Supplementary Material 1 (DOCX 457 KB)

## Data Availability

No datasets were generated or analysed during the current study.
