# Supplementary material for: Beyond Inflammation - A Multidisciplinary Approach to Managing Obesity and Cardiometabolic Risk in Inflammatory Bowel Disease
Source: Curr Diab Rep. 2026 Jun 18;26(1):18. doi: 10.1007/s11892-026-01627-4 (PMC13279483; doi:10.1007/s11892-026-01627-4)
Supplement: Supplementary file 1 — Supplementary Material 1 (DOCX 457 KB) [file 11892_2026_1627_MOESM1_ESM.docx]

Supplementary Information


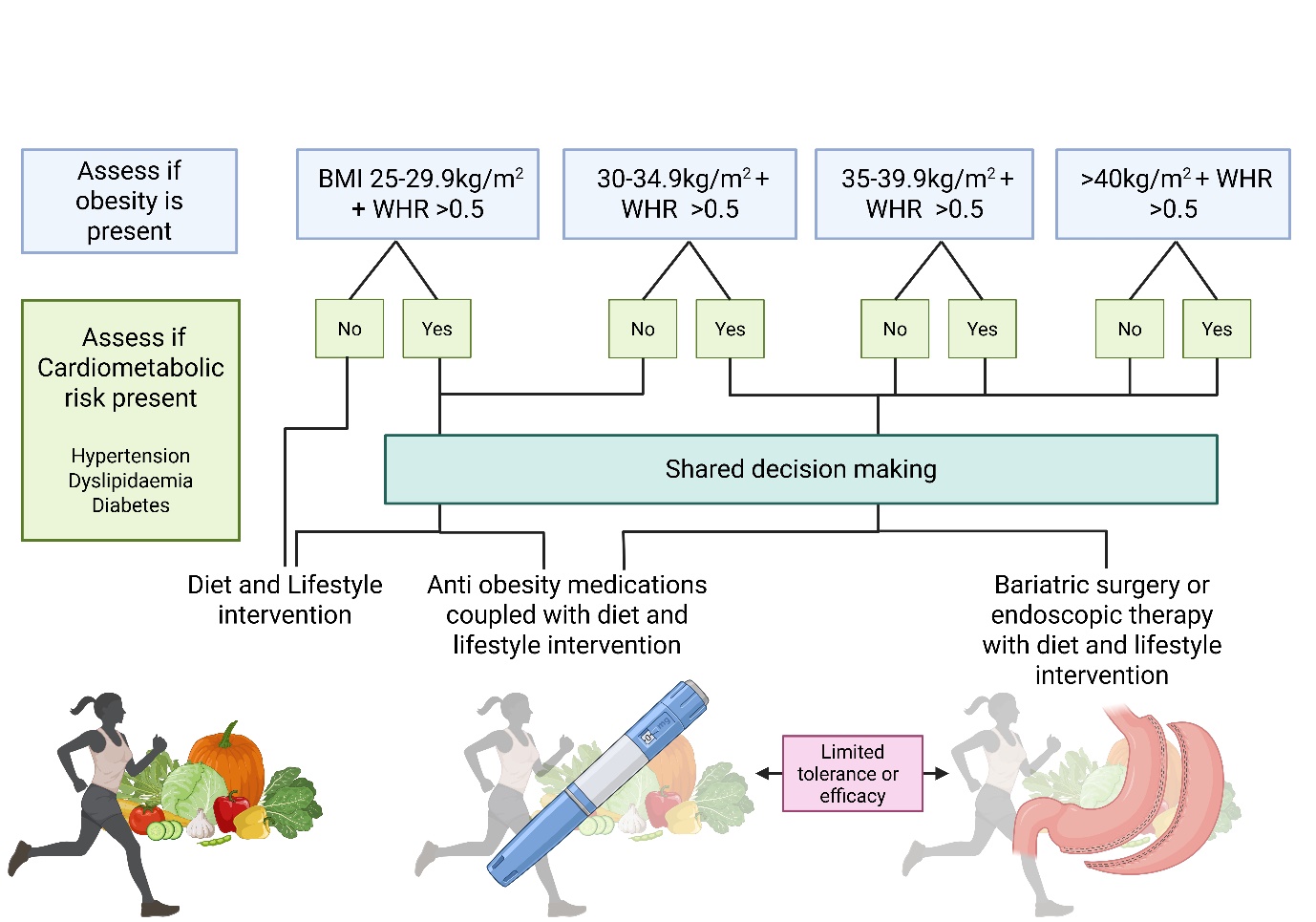


**Supplementary figure 1.** Interdiscipline obesity management treatment algorithm. Abbreviations, BMI, Body mass index; Kilogram, Kg; meter, m; WHR, Waist to height ratio


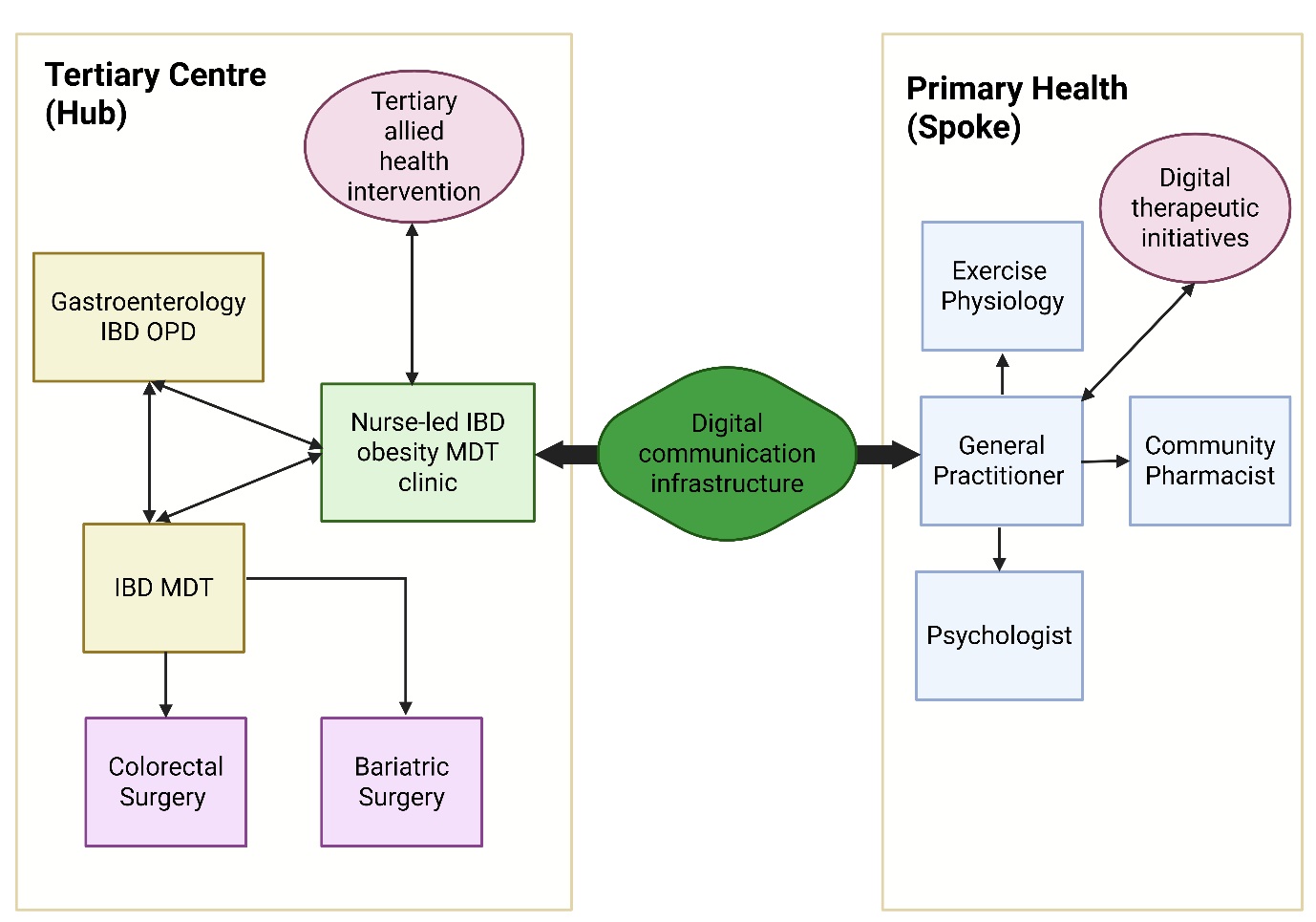


**Supplementary figure 2.** Proposed hub and spoke model of care supported by evidence-based guidelines to manage obese patients with inflammatory bowel disease
